# Inhibition of DNA methyltransferase aberrations reinstates antioxidant aging suppressors and ameliorates renal aging

**DOI:** 10.1111/acel.13526

**Published:** 2021-12-07

**Authors:** Qi Gao, Fang Chen, Lijun Zhang, Ai Wei, Yongxiang Wang, Zhiwei Wu, Wangsen Cao

**Affiliations:** ^1^ Jiangsu Key Lab of Molecular Medicine Nanjing University Medical School Northern Jiangsu People's Hospital Nanjing China

**Keywords:** DNA methylation, epigenetics, KLOTHO, NRF2, renal aging

## Abstract

DNA methylation alterations play mechanistic roles in aging; however, the epigenetic regulators/mediators causally involved in renal aging remain elusive. Here, we report that natural and D‐galactose (D‐gal)‐induced aging kidneys display marked suppression of antiaging factor NRF2 (nuclear factor erythroid‐derived 2‐like 2) and KLOTHO, accompanied by upregulations of DNA methyltransferase (DNMT) 1/3a/3b and NRF2/KLOTHO gene promoter hypermethylations. Administration of a DNMT inhibitor SGI‐1072 effectively hypomethylated the promoters, derepressed NRF2/KLOTHO, and mitigated the structural and functional alterations of renal aging in D‐gal mice. Moreover, oleuropein (OLP), an olive‐derived polyphenol, also displayed similar epigenetic modulation and antiaging effects. OLP inhibited the epigenetic NRF2/KLOTHO suppressions in a gain of DNMT‐sensitive manner in cultured renal cells, demonstrating a strong DNA‐demethylating capacity. In NRF2 knockout and KLOTHO knockdown D‐gal mice, OLP exhibited reduced antiaging effects with KLOTHO displaying a prominent gene effect and effect size; consistently in KLOTHO knockdown mice, the antiaging effects of SGI‐1027 were largely abrogated. Therefore, the KLOTHO recovery is critical for the antiaging effects of DNA demethylation. Collectively, our data indicate that aberrant DNMT1/3a/3b elevations and the resultant suppression of antiaging factors contribute significantly to epigenetic renal aging, which might be targeted for epigenetic intervention by synthetic or natural DNA‐demethylating agents.

## Graphical abstract

Epigenetic cues upregulate DNMT1, DNMT3a, and DNMT3b, resulting in the promoter hypermethylation and expression suppressions of KLOTHO and NRF2, which accelerate D‐gal‐induced renal aging. DNA hypomethylations by DNA‐demethylating agents correct the epigenetic alterations, recover the of KLOTHO and NRF2 losses, and reduce the renal aging alterations.

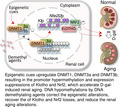

## 1 INTRODUCTION

Development of renal aging is a slow process and manifests as declined renal functions and increased susceptibility to various acute or chronic kidney diseases (Nitta et al., 2013). The aging kidney is histomorphologically characterized by glomerulosclerosis, tubular atrophy, interstitial fibrosis, arteriosclerosis, and loss of cortical mass, and exhibits reduced anti‐oxidative stress potentials and accumulation of inflammatory and fibrotic factors (Abdel‐Rahman & Okusa, 2014). In addition, the aging kidney seems to lose part of its repair ability owing to deficient renal cell proliferation and autophagy, enhanced senescence, and apoptosis (Gekle, 2017), which are attributed to expression changes of many aging‐related genes. Lately, several studies have demonstrated that epigenetic modifications, especially DNA methylation, substantially affect gene expression that are associated with aging (Pal & Tyler, 2016; Zampieri et al., 2015), suggesting an additional control of renal aging process.

DNA methylation, among other epigenetic modifications including protein acetylation/methylation and microRNA interference, is the most stable epigenetic modification that affects the expression of more than 60% of genes (Portela & Esteller, 2010). Three bioactive DNA methyltransferases (DNMT)1, DNMT3a, and DNMT3b add a methyl group from S‐adenosyl‐methionine to the cytosine residue of CpG dinucleotides to form 5‐methylcytosine (5mC) (Maunakea et al., 2010). In the context of gene promoters, hypomethylated CpGs are generally associated with active and constitutively expressed genes, while hypermethylated CpGs correlate with lowly expressed/silenced genes (Bird, 2002). Although genome‐wide association studies and gene‐targeted investigations detect significant changes in DNA methylation with aging and aging‐related kidney diseases in humans and rodents (Unnikrishnan et al., 2019), the direct evidence that alteration of a particular gene expression with its genomic DNA methylation causally affecting renal aging is still lacking.

Oxidative stress is a major pathological factor for premature aging and aging‐associated diseases (Liguori et al., 2018). Past studies have revealed that NRF2 (Nuclear factor erythroid‐derived 2‐like 2, gene *Nfe2l2*) and KLOTHO, two major antiaging factors with antioxidant capacities, are suppressed with aging which correlates with increased incidence of aging‐related kidney disorders (Semba et al., 2011; Semba et al., 2016; Silva‐Palacios et al., 2018). Moreover, NRF2 and KLOTHO deficiencies exacerbate mouse renal aging, while their restorations by a numerous strategies reduce aging phenotype and increase mouse life span (Makoto Kuro‐o et al., 1997; Kurosu et al., 2005; Tarantini et al., 2018), indicating their essential roles in controlling aging processes. NRF2 is a ubiquitously expressed transcriptional factor and a key regulator of redox response. NRF2 positively regulates a number of antioxidant molecules and enzymes by binding to the antioxidant‐response element (ARE) on target gene promoters, thereby elevating the host anti‐oxidative stress and antiaging potentials (Silva‐Palacios et al., 2018). KLOTHO is enriched in distal convoluted tubules in kidney (Azuma et al., 2012) and exists as both a transmembrane and a secreted protein known to exert the antiaging functions mainly by inhibiting insulin/insulin‐like growth factor‐1 (IGF‐1) signaling, excessive inflammation, and oxidative stress (Liu et al., 2011; Masuda et al., 2005). The promoters of both NRF2 gene (*Nfe2l2*) and KLOTHO gene (*Kl*) contain typical CpG islands and their suppressions due to DNA methylation aberrations are reported in various aging‐related disorders (Reyes‐Aguirre & Lamas, 2016; Zhang et al., 2017); however, whether the suppressions involve aberrant DNA methylation modification mechanistically relevant to renal aging remains to be determined.

In this study, we investigated the altered DNA methylation modifications of NRF2 and KLOTHO expression in kidneys of natural and D‐galactose (D‐gal)‐treated accelerated aging mice (Azman & Zakaria, 2019). We observed that both NRF2 and KLOTHO were markedly suppressed, which correlated with their promoter hypermethylations and aberrant DNMT1/3a/3b elevations. We then assessed a synthetic DNA‐demethylating agent SGI‐1072 and a small compound oleuropein (OLP) found in natural olive for their epigenetic regulations of the KLOTHO/NRF2 suppression and anti‐renal aging efficacies. Our data might reveal important epigenetic characters of renal aging and provide novel insights into potential prophylactic and therapeutic anti‐renal aging strategies.

## 2 RESULTS

### 2.1 KLOTHO expression is suppressed in natural and accelerated mouse aging kidney

**FIGURE 1:**
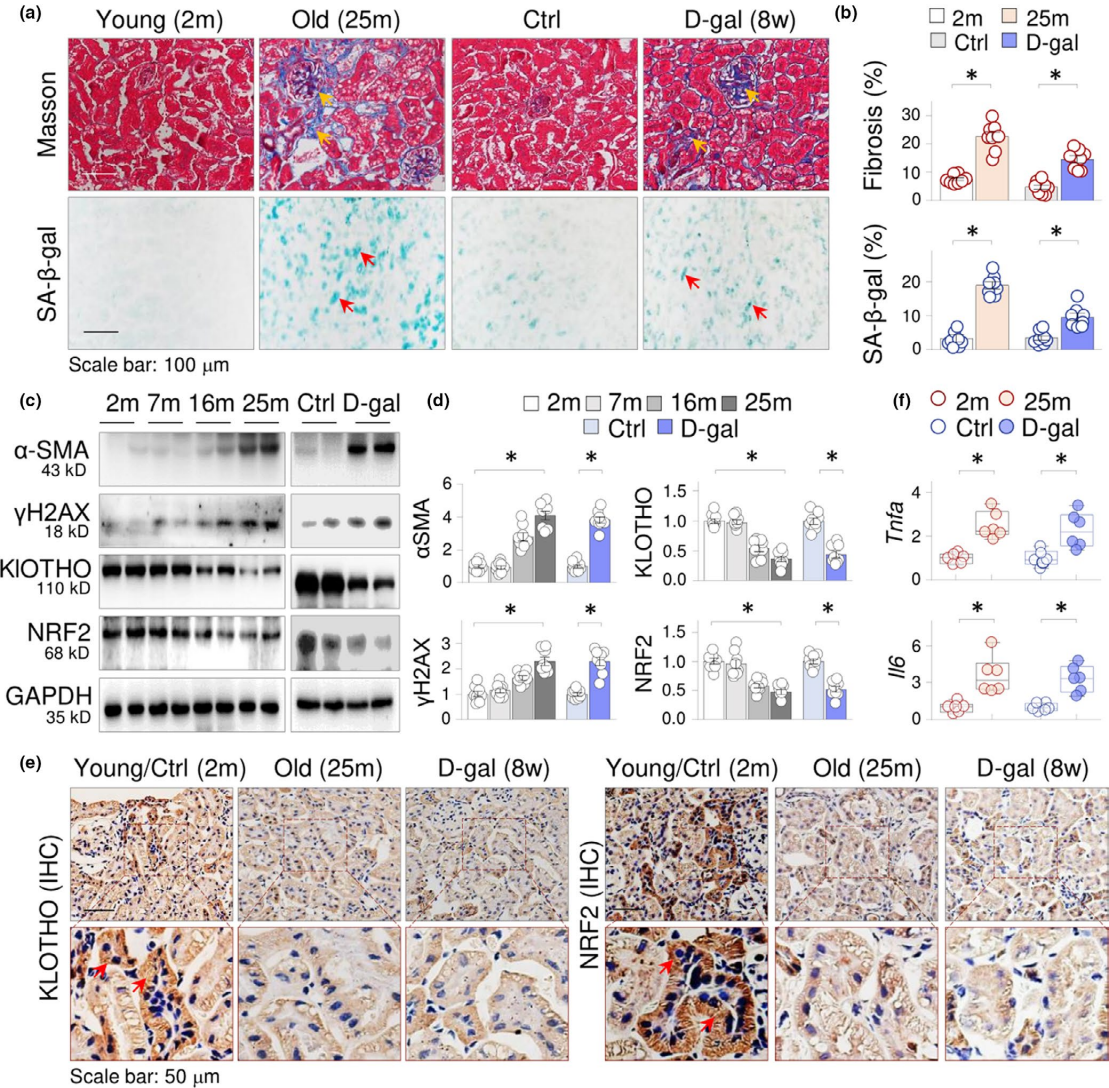
KLOTHO expression is suppressed in natural and accelerated mouse aging kidney. (a) Representative photomicrographs of kidney sections of young (2 months), old mice (25 months), D‐gal‐injected (8 weeks) and the control mice (n = 8 per group) stained by Masson's trichrome staining (the yellow arrows indicated the fibrotic collagen deposition) or senescence‐associated β‐galactosidase staining (the red arrows indicate the β‐gal‐positive cells). (b) Quantifications of renal fibrosis and β‐galactosidase staining. Data were presented as the percentages of fibrotic or β‐gal‐positive areas over the respective total areas (each dot in the bar represented the average of 10 fields of view from one animal within the group). (c) Western blots of renal tissues for α‐SMA, γH2AX, KLOTHO, and NRF2 from 2, 7, 15, and 25 m mice, as well as from the control and D‐gal‐injected mice (8 weeks). GAPDH served as internal loading control. Two samples from each group were shown. (d) Quantitation of (c). The values were presented as means ±SEM. *p < 0.05, one‐way ANOVA. (e) Representative renal sections of mice in (a) stained by Immunohistochemistry (IHC) for KLOTHO and NRF2. The images at lower panel were the amplified frames in the corresponding upper panel. The arrows indicated positively stained tubular cells. (f) qRT‐PCR of the renal tissues for TNF‐α and IL‐6 mRNAs. Data were presented as box‐and‐whisker plots. *p < 0.05, Student's *t* test

We first examined the protein expression of KLOTHO and NRF2 in mouse aging kidneys. The kidneys from both natural aging mice (25 months old, n = 8 per group) and an accelerated mouse aging model induced by D‐galactose (D‐gal) injection (n = 8 per group, 8 weeks) displayed increased collagen depositions in renal sections, especially in glomerulus, as demonstrated by Masson's trichrome staining (Figure 1a and b, the upper panels, indicated by arrows), as well as SA‐β‐galactosidase (SA‐β‐gal) staining (Figure 1a and b, the lower panels, indicated by arrows). Notably, the natural aging kidney (25 m) showed more fibrotic collagen depositions (Figure 1a and b, comparing panel 2 and 4). Moreover, the natural aging kidney displayed time‐dependent increases (2, 7, 16, and 25 months) of myofibroblast marker α‐SMA and aging‐associated DNA double‐strand break marker γH2AX (phosphorylated histone H2AX), as well as decreased KLOTHO and NRF2 (Figure 1c and d, the left panels). The similar protein expression alterations were also observed in D‐gal‐treated aging kidneys (Figure 1c and d, the right panels). We also examined kidney sections by immunohistochemical staining and confirmed that both KLOTHO and NRF2 were enriched in renal distal tubular epithelial cells in young control mice, but the levels were noticeably reduced in old (25 m) and D‐gal‐treated aging mice (8 weeks, Figure 1e). In addition, both natural and D‐gal‐induced aging kidneys displayed inductions of inflammatory cytokines TNF‐α and IL‐6 (Figure 1f). These results clearly demonstrate that suppressions of antiaging factor KLOTHO and NRF2 are features of renal aging.

### 2.2 Aging kidneys exhibit KLOTHO gene promoter hypermethylation and aberrant DNMT1/3a/3b elevations

**FIGURE 2:**
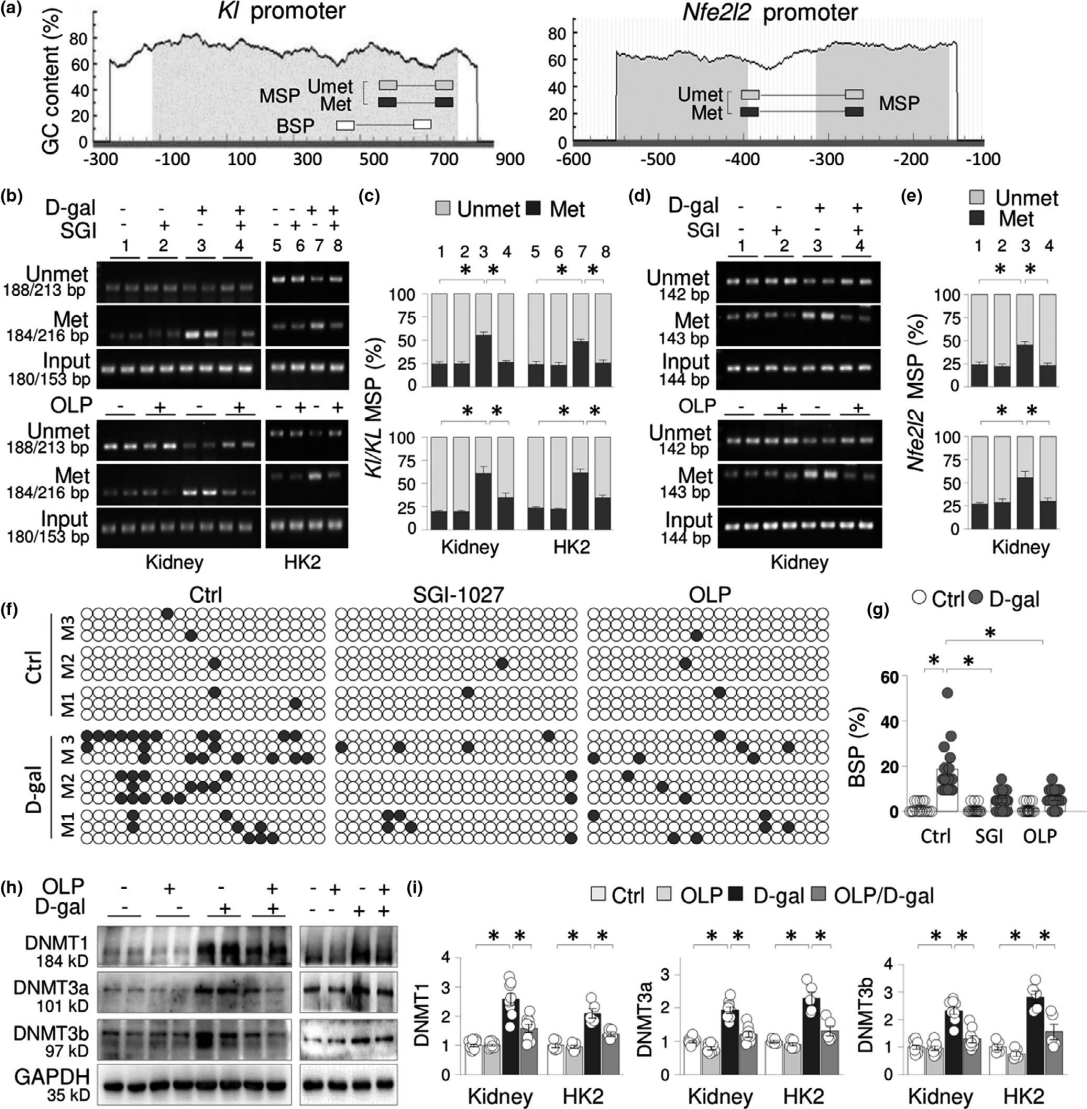
Aging kidneys exhibit KLOTHO gene promoter hypermethylation and aberrant DNMT1/3a/3b elevations. (a) Schematic diagrams of mouse *Kl* and *Nfe2/2* promoters. The positions of CpG islands (gray area) and MSP/BSP primers were depicted relative to the transcription‐starting site. (b and d) MSP analysis of renal tissues of mice (8 weeks, the left) or HK2 cells (48 h, the right) treated with vehicle, D‐gal (100 mM), SGI‐1027 (SGI, (10 μM, the upper panel)/OLP (50 μM, the lower panel), or SGI‐1027/OLP plus D‐gal. Representative agarose gel analysis of methylated (Methyl), unmethylated (Unmet) and input PCR products for (b) Mouse tissue *Kl*/human HK2 cell *KL* or (d) *Nfe2l2*. (c and e) Quantifications for (b; c) or (d; e). Values were presented as mean percentages ±SEM after adjusted with input PCR products, *p < 0.05, Two‐way ANOVA. (f) BSP analysis of kidney tissues of mice from Figure 3a for *Kl* promoter. For each group, 3 randomly selected mice were analyzed (M1, M2, and M3 were shown). After PCR, 5 clones from each animal were sequenced, of which three clones were presented and additional two clones were presented as Figure S2. One box represented one mouse. Each row of dots in the boxes represented one single sequenced clone, and each dot represented one CpG site. Empty or dark dots indicated unmethylated or methylated CpGs, respectively. (g) Quantification of (f). Data are presented as ±SEM. Each circle represented the percentage of methylated CpGs over total CpGs of all 15 cloned fragments from 3 animals in each group. *p < 0.05, two‐way ANOVA. (h) Western blots of renal tissues of mice (the left panel) or HK2 cells (the right panel) treated with vehicle, OLP, D‐gal, or both as in Figure 3a for DNMT1(Dt1), DNMT3a (Dt3a), and DNMT3b (Dt3b). Two random samples from kidneys and one representative sample from cell assay were shown. (i) Quantitation of (h). The values were presented as means ±SEM. *p < 0.05, two‐way ANOVA

To explore the possibility that aberrant DNA methylation modification might cause the KLOTHO and NRF2 suppressions, we first analyzed the promoters of both mouse KLOTHO gene (*Kl*) and NRF2 genes (*Nfe2l2*) by online software MethPrimer (http://www.urogene.org/methprimer). Both *Kl* and *Nfe2l2* promoters contain typical CpG islands located at −110/800 (*Kl*) and −550/−150 (*Nfe2l2*) loci, respectively (Figure 2a). We then examined the promoter DNA methylation status of renal tissues from natural (2, 16, and 25 m) and D‐gal‐treated aging mice (8 weeks), and found that the promoter methylation levels of *Kl* (594/777) increased from 23.11% ± 1.76% of 2 m mice to 69.47% ± 4.88% of 25 m mice (*p* < 0.05) and from 24.92% ± 1.37% of control to 49.29% ± 5.16% of D‐gal mice (*p* < 0.05), respectively. In addition, the promoter methylation of *Nfe2l2* (−389/−265 locus) increased from 23.55% ± 4.36% of 2 m mice to 68.37% ± 5.84% of 25 m mice (*p* < 0.05) and from 26.46% ± 6.79% of control to 56.17% ± 5.11% of D‐gal mice (*p* < 0.05), respectively (Figure S1A and B). Since DNA methylation is positively regulated by DNMT, we further assessed the renal protein levels of three bioactive DNMTs and found that DNMT1 started to increase on 7th month, and DNMT3a and DNMT3b also increased later (16th and 25th months) in natural aging and D‐gal‐treated (8 weeks) mice (Figure S1C and D). These results indicate that the increased DNMT1/3a/3b expression might be responsible for the promoter hypermethylation and the suppression of KLOTHO and NRF2 in aging kidneys.

To confirm that the KLOTHO and NRF2 suppressions are due to the DNMT aberrations, we tested whether SGI‐1027, a quinoline‐based DNMT inhibitor with IC_50_s of 12.5 μM, 8 μM and 7.5 μM, for DNMT1, DNMT3a, and DNMT3b (Datta et al., 2009), inhibits the epigenetic alterations. The results showed that SGI‐1072 administration effectively demethylated *Kl*/*KL* promoters from 55.45% ± 3.45% of D‐gal‐treated kidney to 26.2% ± 1.82% (*p* < 0.05), and from 48.71% ± 2.25% of D‐gal‐treated HK2 cells to 25.18% ± 3.38% (*p* < 0.05, Figure 2b and c, the upper panel), and *Nfe2l2* promoter from 44.95% ± 4.48% of D‐gal‐treated kidney to 22.81% ± 2.58% (*p* < 0.05, Figure 2d and e, the upper panel), supporting that the promoter hypermethylations of both *Kl* and *Nfe2l2* in D‐gal mouse kidneys are mainly caused by aberrant DNMT1/3a/3b elevations.

SGI‐1027 is a synthetic DNMT inhibitor. Because synthetic epigenetic drugs, such as decitabine (5‐Aza‐2′‐deoxycytidine), might be potentially cytotoxic, and also because many dietary or medicinal plant components, especially polyphenols, possess antiaging and epigenetic modulating activities with tolerable side effects, we also tested OLP, a polyphenol isolated from olive leaves with antiaging capacity (Leri et al., 2020), for its demethylating and anti‐renal aging potencies. Similarly, we treated D‐gal‐treated mice and HK2 cells with OLP and found that OLP effectively demethylated the *Kl*/*KL* promoters from 61.92% ± 7.7% of D‐gal‐treated kidney to 35.09% ± 4.55% (*p* < 0.05), and from 61.39% ± 4.38% of D‐gal‐treated HK2 cells to 34.82% ± 2.83% (*p* < 0.05, Figure 2b and c, the lower panels), and the *Nfe2l2* promoter from 55.68% ± 7.19% of D‐gal‐treated kidney to 30.19% ± 3.17% (*p* < 0.05, Figure 2d and e, the lower panels). We also analyzed the *Kl* promoter methylation of the same locus by BSP, the gold standard for DNA methylation assessment. The *Kl* promoter region analyzed (466/700) contained 21 CpG sites and displayed increased methylation in D‐gal‐treated kidney from 1.58% ± 0.59% to 18.73% ± 3.06% (*p* < 0.05), whereas SGI‐1027 and OLP treatments reduced the level to 4.76% ± 1.14% and 6.03% ± 1.09%, respectively (*p* < 0.05, Figure 2f,g and Figure S2). Further, the results from Western blotting showed that OLP treatment significantly lowered the elevated DNMT1/3a/3a in D‐gal‐treated kidneys (Figure 2h and i), suggesting that OLP possesses strong DNA‐demethylating capacity.

### 2.3 SGI‐1027 and OLP derepress KLOTHO and reduce renal aging in D‐gal‐treated mice

**FIGURE 3:**
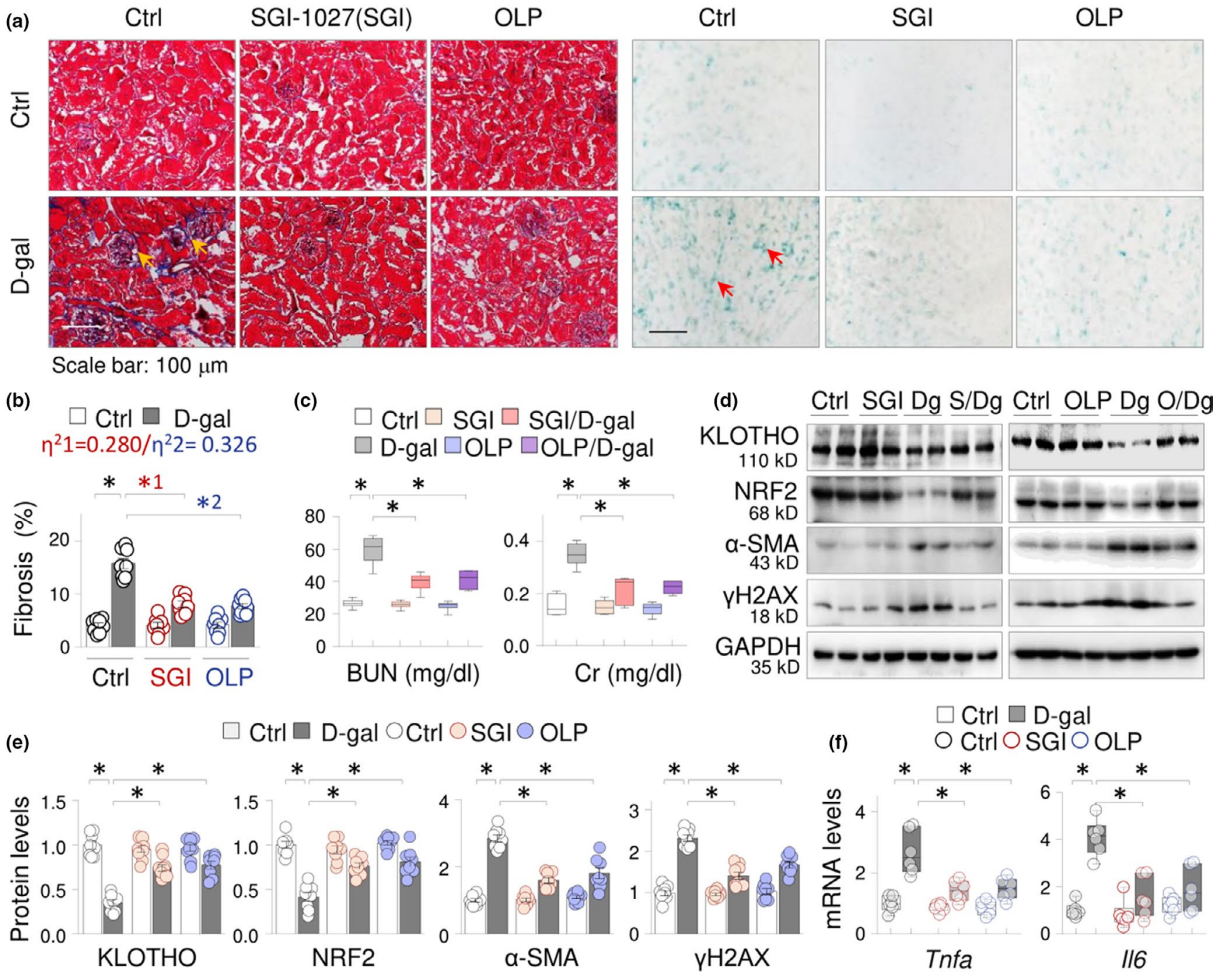
SGI‐1027 and Oleuropein (OLP) derepress KLOTHO and reduce renal aging pathologies in D‐gal‐incurred aging mice. The vehicle control or D‐gal‐injected ICR mice were treated with or without SGI‐1027 or OLP for 8 weeks (8 mice in each group). (a) Representative photomicrographs of kidney sections stained by Masson's trichrome staining (the left half, the arrows indicated the fibrotic collagen deposition) or senescence‐associated β‐gal staining (the right half, the arrows indicated the β‐gal‐positive cells). (b) Quantifications of renal fibrosis. Data were presented as means ±SEM. *p < 0.05, two‐way ANOVA. The effect sizes of interaction between D‐gal treatment and SGI‐1027 (η^2^1) or OLP (η^2^2) on fibrotic deposition were indicated. (c) Serum levels of blood urea nitrogen (BUN) and creatinine (Cr). Data were presented as box‐and‐whisker plots. *p < 0.05, two‐way ANOVA. (d) Western blots of the renal tissues for KLOTHO, NRF2, α‐SMA, and γH2AX. Two samples from each group were shown. (e) Quantitation of Figure 3d. The values were presented as means ±SEM. *p < 0.05, two‐way ANOVA. (f) qRT‐PCR of renal tissues for *Tnfa* and *Il6* mRNAs. Data were presented as box‐and‐whisker plots. *p < 0.05, two‐way ANOVA

To test whether the demethylation of *Kl* and *Nfe2l2* promoters by SGI‐1027 and OLP affects their expression and the functional relevancies to renal aging, we treated D‐gal mice with SGI‐1027 or OLP separately (n = 8 per group, 8 weeks). As anticipated, the kidney sections from D‐gal mice showed increased collagen depositions in interstitium and glomerulus areas (Figure 3a, the left panel) and β‐gal‐positive cells (Figure 3a, the right panel). SGI‐1027 and OLP treatments significantly reduced the depositions from 15.73% ± 0.97% to 7.69% ± 0.51% (*p* < 0.05) and to 8.07% ± 0.59% (*p* < 0.05), respectively (Figure 3b), as well as the numbers of β‐gal‐positive cells. Since aging kidneys are featured by infiltrated macrophages, which also express β‐gal, we also stained the sections for macrophage marker CD68 and found that CD68‐positive macrophages appeared in D‐gal‐treated kidney, but largely disappeared after SGI‐1027 or OLP treatment (Figure S3). These macrophages only accounted for a small portion of β‐gal‐positive cells, suggesting that renal aging occurs mainly in kidney parenchymal cells. We further calculated the effect sizes of SGI‐1027(η^2^1) and OLP (η^2^2) on the fibrosis severities, which were η^2^1 = 0.280 and η^2^2 = 0.326 (Figure 3b, the insert), respectively, indicating that both SGI‐1027 and OLP effectively reduce the fibrosis intensities with OLP showing a stronger capacity. We also found that serum levels of creatinine (Cr) and blood urea nitrogen (BUN), two key parameters of renal functions, increased in D‐gal‐treated mice, which were significantly lowered by SGI‐1027 or OLP treatment (Figure 3c). Moreover, SGI‐1027 and OLP treatments inhibited the abnormal renal expression of KLOTHO, NRF2, α‐SMA, and γH2AX proteins (Figure 3d and e) and TNF‐α (*Tnfa*) and IL‐6 (*Il6*) mRNAs in D‐gal‐treated mice (Figure 3f). Since KLOTHO and NRF2 inhibit aging, these results suggest that the preservations of KLOTHO and NRF2 by SGI‐1027 or OLP might contribute to their anti‐renal aging functions.

### 2.4 OLP preservation of KLOTHO is sensitive to gain of DNMT function in renal cells

**FIGURE 4:**
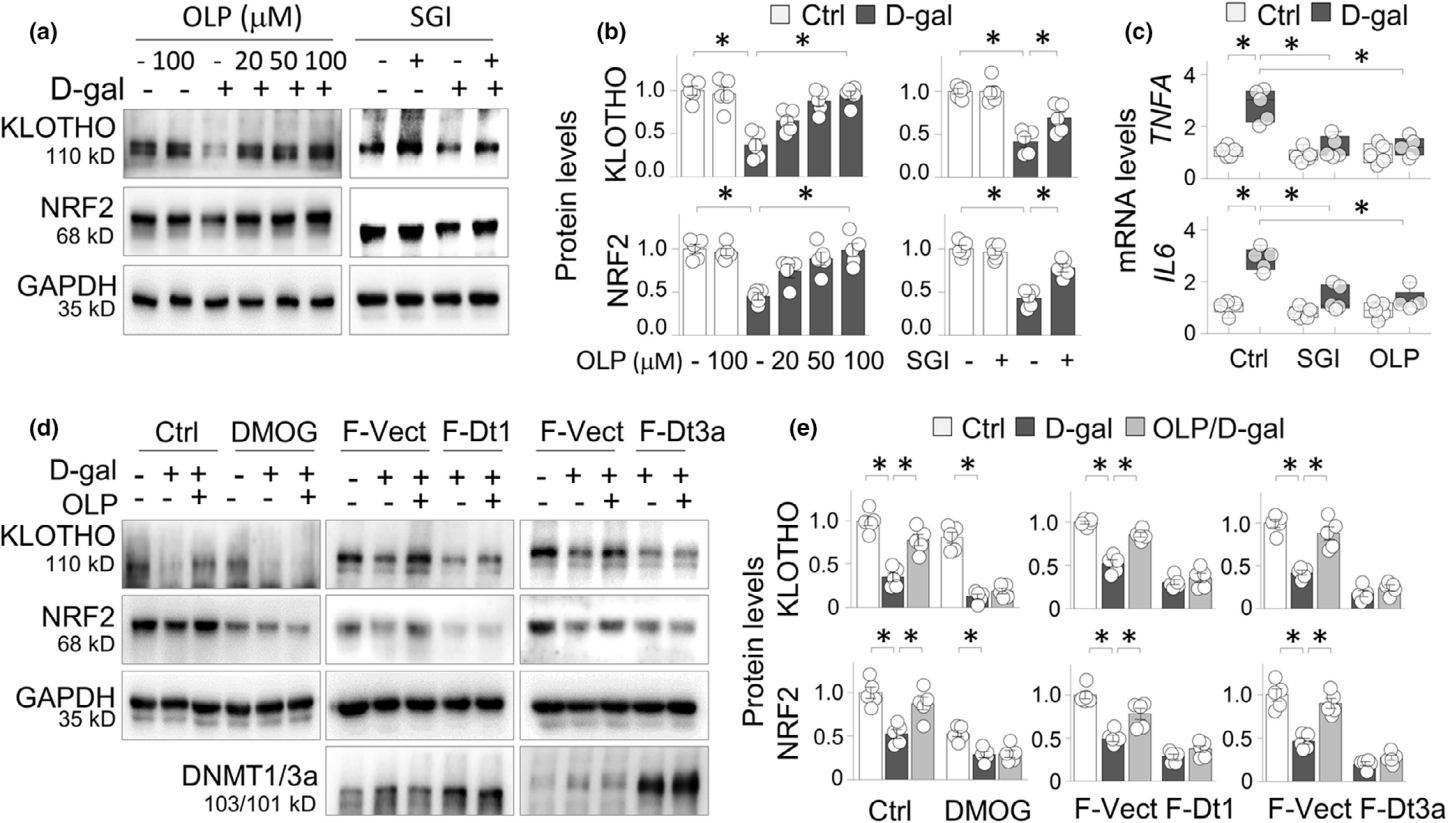
OLP preservation of KLOTHO is sensitive to gain of DNMT activities in renal cells. (a) Western blots of KLOTHO and NRF2 expression from HK2 cells treated with vehicle or D‐gal (100 mM) in absence or presence of increasing doses of OLP (20, 50, and 100 μM) or SGI‐1027 (SGI, 10 μM) for 60 h. (b) Quantifications of (a). Data are presented as means ±SEM; *p < 0.05, two‐way ANOVA. (c) qRT‐PCR of *TNFA* and *IL6* mRNAs from HK2 cells treated with control and D‐gal (100 mM) in absence or presence of SGI (10 μM) or OLP (50 μM) for 24 h. Data were presented as box‐and‐whisker plots of 5 repeated experiments. *p < 0.05, three‐way ANOVA (d) Western blots of KLOTHO, NRF2, DNMT1, and DNMT3a from HK2 cells treated with D‐gal (100 mM) or/and OLP (50 μM) in absence or presence of DMOG (20 μM, the left panel), over‐expressed flag‐tagged DNMT1 (fDt1, the middle panel) or flag‐tagged DNMT3a (fDt3a, the right panel) for 60 h. (e) Quantification of (d). Data are presented as means ±SEM based on 5 independent experiments. *p < 0.05, two‐way ANOVA with Tukey's post hoc test

OLP displays multiple pharmacological activities. We confirmed that OLP reversed the D‐gal‐induced KLOTHO and NRF2 suppressions in HK2 cells in a dose‐dependent manner, similarly to that of SGI‐1027 (Figure 4a and b). We further found that both SGI‐1027 and OLP effectively reduced the induction of *TNFA* and *IL6* mRNAs in D‐gal‐treated HK2 cells (Figure 4c), suggesting that the KLOTHO/NRF2 restoration by DNMT inhibition mitigated the inflammatory response, and renal epithelial cells contributed to the production of inflammatory cytokines. To assess whether OLP preservations of KLOTHO and NRF2 are due to its inhibition of the DNMT elevations, we tested whether gain of DNMT function affects the OLP restoration of KLOTHO and NRF2 by two strategies. We first pre‐treated HK2 cells with or without DMOG (dimethyloxallyl glycine), a small molecule of TET enzyme inhibitor (Amouroux et al., 2016) that supposedly counteracts the activity of DNMT inhibition (Hideyuki Takeshima et al., 2020), and found that DMOG treatment blocked OLP alleviation of the KLOTHO and NRF2 suppressions induced by D‐gal (Figure 4d and e, the left panel). Further, overexpression of flag‐tagged DNMT1 or DNMT 3a diminished OLP recovery of the KLOTHO and NRF2 losses, respectively (Figure 4d and e, the middle and right panels). These data strongly support that a considerable portion of the OLP effects on KLOTHO and NRF2 suppressions in aging kidney is due to its inhibition of the aberrant DNMT elevations.

### 2.5 KLOTHO is critical for the anti‐renal aging effects of OLP

**FIGURE 5:**
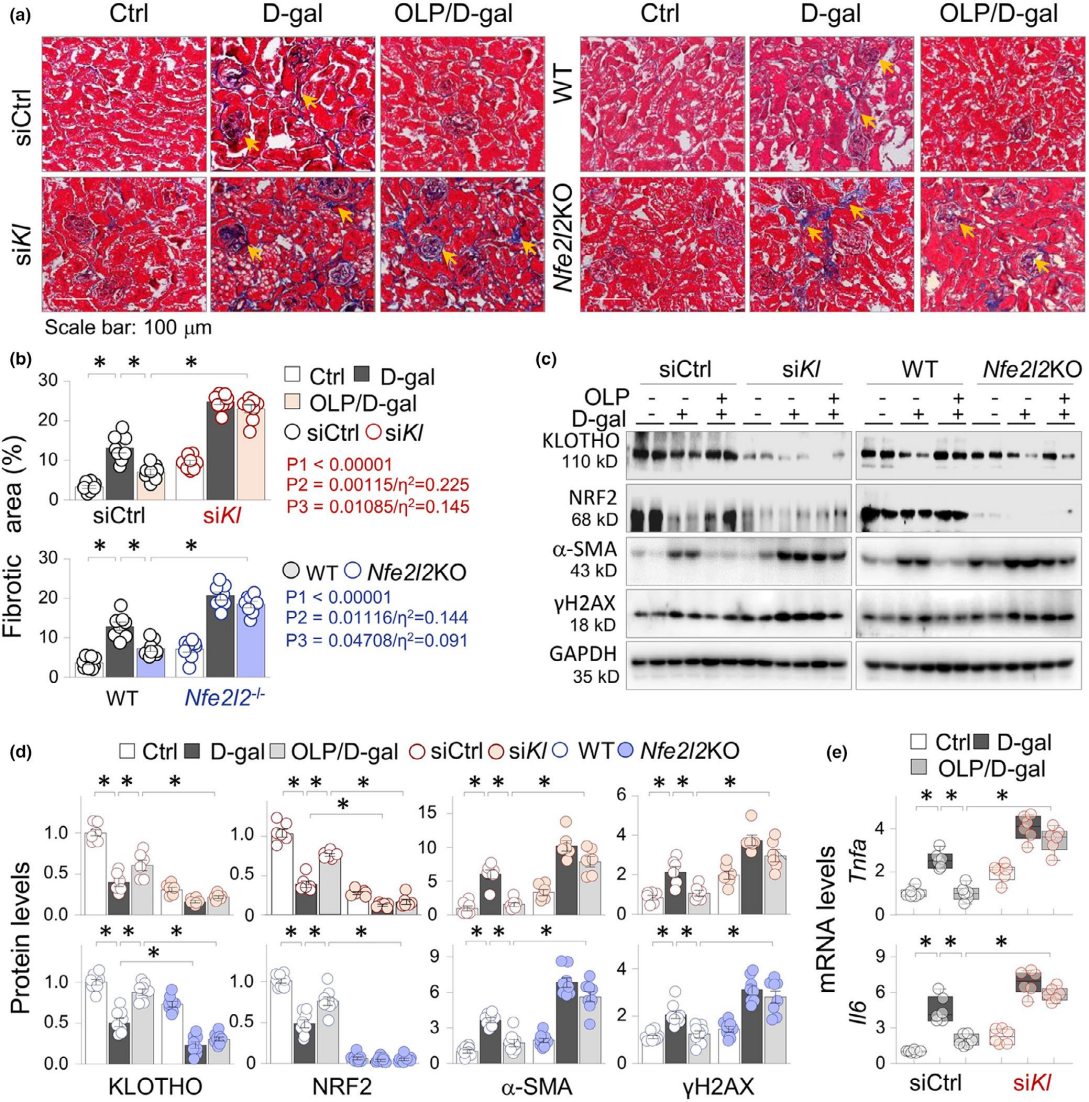
KLOTHO preservation is critical for the anti‐renal aging effects of OLP. Mice receiving siRNA‐Control (siCtrl) or siRNA‐KLOTHO (si*Kl*, n = 8), or *Nfe2l2*WT and *Nfe2l2*KO mice (n = 8) were grouped into vehicle control, D‐gal, and OLP/D‐gal mice as before (8 weeks). (a) Representative photomicrographs of kidney sections (Masson's trichrome staining) for siCtrl/si*Kl* mice (the left panel) and *Nfe2l2*WT/KO mice (the right panel). The arrows indicated fibrotic collagen deposition areas. (b) Quantitation of renal fibrotic areas of siCtrl/si*Kl* (the upper panel) and *Nfe2l2*WT/KO mice (the lower panel) in (a). The effects of *Kl* and *Nfe2l2* genotypes (P1), the effects of interactions between *Kl* or *Nfe2l2* genotype and D‐gal treatment (P2), or the interactions between *Kl* or *Nfe2l2* genotype and OLP intervention (P3), as well as their corresponding effect sizes were indicated. (c) Western blots. The renal tissues from experimental mice in (a) were assayed for KLOTHO, NRF2, α‐SMA, and γH2AX. Two samples from each group were shown. (d) Quantification of (c). Data were presented as means ±SEM. *p < 0.05, three‐way ANOVA followed by Tukey's post hoc test. (e) qRT‐PCR of the renal tissues from siCtrl and si*Kl* mice (n = 6) for *Tnfa* and *Il6* mRNAs. Data were presented as box‐and‐whisker plots. *p < 0.05, three‐way ANOVA followed by Tukey's post hoc test

To further assess the role of KLOTHO and NRF2 preservation by OLP in renal aging, we compared the renal‐protective effects of OLP between KLOTHO knockdown (si*Kl*) and the control mice (siCtrl, n = 6 per group), as well as between *Nfe2l2*WT and *Nfe2l2*KO mice (n = 8 per group). As revealed by the renal fibrotic scoring, si*Kl* mice displayed more collagen depositions than siCrtl mice under control condition (9.44% ± 1.69% vs. 3.3% ± 1.16% of siCtrl mice, (*p* < 0.05, Figure 5a and b, the left and the upper panels), while the fibrotic extents *of Nfe2l2*KO and *Nfe2l2*WT control mice were similar (Figure 5a and b, the right and lower panels). After D‐gal treatments, both si*Kl* and *Nfe2l2*KO mice showed significant increases of fibrotic depositions than their control littermates (24.79% ± 2.0% vs. 13.09% ± 3.3% of siCtrl D‐gal mice, *p* < 0.05; and 20.63% ± 2.82% vs. 12.96% ± 3.0% of *Nfe2l2*WT D‐gal mice, *p* < 0.05). OLP treatments effectively reduced the fibrotic areas in siCtrl and *Nfe2l2WT* D‐gal mice (Figure 5b, comparing the 2nd and 3rd columns); however, the inhibitions were significantly reduced in si*Kl* or *Nfe2l2*KO D‐gal mice (Figure 5a and b, the 3rd and 6th columns). Since si*Kl* control mice showed spontaneous renal fibrotic alterations and si*Kl* D‐gal mice developed more severe fibrotic depositions, we calculated the group main effect of genotype (P1), effect of group interactions between genotype and D‐gal treatment (P2) and between genotype and OLP intervention (P3, Figure 5b, the inserts), and the corresponding effect size (η^2^). The results showed that the renal fibrotic alterations were significantly affected by *Kl* and *Nfe2l2* genotypes (*p *< 0.00001), and by interactions between *Kl* and D‐gal treatment (P2 = 0.00115, η^2^ = 0.225) and between *Kl* and OLP intervention (P3 = 0.01085, η^2^ = 0.145), which were greater than that of the interactions between *Nfe2l2* and D‐gal treatment (P2 = 0.01116, η^2^ = 0.144) and between *Nfe2l2* and OLP intervention (P3 = 0.04708, η^2^ = 0.091). Consistently, OLP effectively normalized the adverse expression of KLOTHO, NRF2, α‐SMA, and γH2AX (Figure 5c and d), as well as the induction of *Tnfa* and *Il6* (Figure 5e) in siCtrl and WT D‐gal mice, but the effects were largely obligated in si*Kl* and *Nfe2l2*KO D‐gal mice. Taken together, these results suggest that KLOTHO affects renal aging more than NRF2 and the derepression of KLOTHO by OLP provides stronger anti‐renal aging effects than that of NRF2.

### 2.6 KLOTHO deficiency abrogates the anti‐renal aging effects of SGI‐1027

**FIGURE 6:**
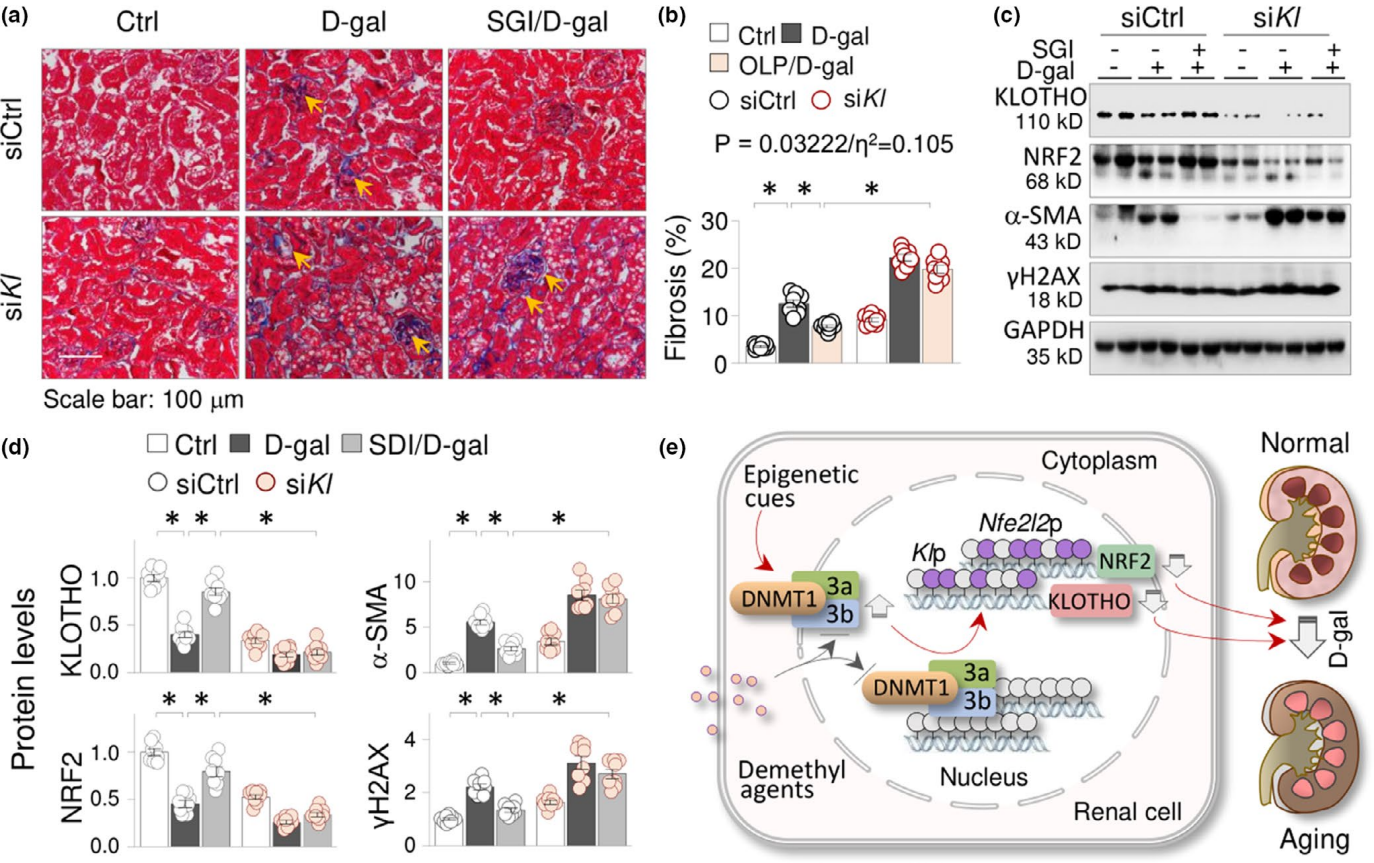
KLOTHO deficiency abrogates the anti‐renal aging effects of SGI‐1027. Mice receiving siRNA‐Control (siCtrl) or siRNA‐KLOTHO (*siKl*) were grouped into vehicle control, D‐gal, and SGI‐1027/D‐gal mice as before (n = 8 per group, 8 weeks). (a) Representative photomicrographs of kidney sections (Masson's trichrome staining) for siCtrl/si*Kl* mice. The arrows indicated fibrotic collagen deposition areas. (b) Quantitation of renal fibrotic areas of (a). The effect (P) and the effect size (η^2^) of interaction between *Kl* genotype and SGI‐1027 intervention were indicated. (c) Western blots. The renal tissues from experimental mice in (a) were assayed for KLOTHO, NRF2, α‐SMA, and γH2AX. Two samples from each group were shown. (d) Quantification of (c). Data were presented as means ±SEM. *p < 0.05, three‐way ANOVA followed by Tukey's post hoc test. (e) A schematic diagram of DNMT1/3a/3b elevations and the resultant KLOTHO suppression and renal aging. Epigenetic cues upregulate DNMT1, DNMT3a, and DNMT3b, resulting in the promoter hypermethylation and expression suppressions of KLOTHO and NRF2, which accelerate D‐gal‐induced renal aging. DNA hypomethylations by DNA‐demethylating agents correct the epigenetic alterations, recover the repressions of KLOTHO and NRF2 losses, and reduce the renal aging

To further prove that the KLOTHO suppression due to aberrant DNA methylation is a major causative factor of renal aging, we compared the antiaging effects of SGI‐1027 between si*Kl* and the control mice (siCtrl, n = 8 per group). The results confirmed that SGI‐1027 effectively minimized the renal fibrotic areas in siCtrl D‐gal mice (7.76% ± 0.29% vs. 12.58% ± 0.75% D‐gal mice, *p* < 0.05); however, the effects were largely reduced in si*Kl* mice (7.76% ± 0.29% siCtrl vs. 19.82% ± 0.82% si*Kl*, *p* < 0.05. Figure 6a and b). We also calculated the effect of interaction between genotype and SGI‐1027 intervention (*p* = 0.03222, Figure 6b, the insert), and the corresponding effect size (η^2^ = 0.105), which were smaller than that of OLP (*p* = 0.01085/η^2^ = 0.145). Moreover, SGI‐1027 effectively corrected the abnormal expression of KLOTHO, NRF2, α‐SMA, and γH2AX in siCtrl D‐gal mice, but the effects were significantly reduced in si*Kl* D‐gal mice (Figure 6c and d). Collectively, these results not only suggest that KLOTHO suppression due to DNA hypermethylation causally affects renal aging, but also support that OLP preservation of KLOTHO via DNA demethylation plays a significant role in its anti‐renal aging activities.

## 3 DISCUSSION

In this study, we made several meaningful findings toward a better understanding of the epigenetic mechanisms of renal aging. (1) KLOTHO and NRF2, two antiaging factors with antioxidant properties, were suppressed in aging kidneys due to DNMT1/3a/3b elevation‐incurred transcriptional inhibition; (2) DNA‐demethylating agent SGI‐1027 and OLP effectively reduced the epigenetic losses of KLOTHO and NRF2, and mitigated the D‐gal‐induced renal aging alterations; (3) OLP possesses strong DNMT‐inhibiting capabilities, as it effectively lowered the elevated DNMT1/3a/3b in aging kidneys; (4) In KLOTHO gene knockdown and NRF2 gene knockout mice, NRF2 and KLOTHO were mutually repressed and the antiaging effects of OLP were significantly abrogated, suggesting that KLOTHO and NRF2, and possibly their mutual regulations, are crucial in the development of renal aging; (5) KLOTHO had a greater genotype effect and effect size of interaction between KLOTHO genotype and OLP intervention comparing to that of NRF2, and KLOTHO knockdown in mice significantly reduced the anti‐renal aging effects of DNMT inhibition by SGI‐1027. Thus, our study demonstrated that aberrant DNMT1/3a/3b elevations and the resultant suppressions of KLOTHO and NRF2 contribute significantly to renal aging, which can be effectively targeted by epigenetic intervention with synthetic or natural DNA‐demethylating agents (Figure 6e).

Demonstrating the causative role of aberrant DNMT1/3a/3b elevations in aging kidney is an important discovery of our study. Past studies have established that aging is accompanied by a global decrease in DNA methylation in aging humans (Bjornsson, 2008) and mice (Singhal et al., 1987). However, DNA methylation modifications occur in a tissue and/or gene‐specific manner and loss of DNA methylation of genome‐wide is accompanied by a gain of methylation in CpG islands in or near gene promoters (van Otterdijk et al., 2013). DNMT inhibitor 5‐Azacytidine reverses the aging phenotypes of mesenchymal stem cells (Kornicka et al., 2016), suggesting that gain of DNMT function promotes aging. However, DNA methylation status is affected by multiple regulatory proteins, including DNA insulator proteins, DNMTs, methyl‐CpG‐binding protein, and TET (ten‐eleven translocation) enzymes (Unnikrishnan et al., 2019). The information regarding the DNMT expression in aging kidney is limited, except that one study reported upregulated DNMT1 and DNMT3a (Pushpakumar et al., 2020). We found that DNMT1/3a/3b were all upregulated in natural and D‐gal aging kidneys, which caused hypermethylation of KLOTHO and NRF2 gene promoters and renal aging, as DNA‐demethylating agents corrected the epigenetic aberrations and improve the renal aging alterations in a KLOTHO and NRF2‐dependent fashion. These observations provide a solid basis to further investigate whether the downregulation of other antiaging molecules is affected by the similar DNMT alterations in aging kidney or extrarenal organs.

One intriguing observation of our study is that both KLOTHO and NRF2 are repressed in aging kidneys and mutually inhibited during renal aging as demonstrated in KLOTHO gene knockdown or NRF2 gene knockout mice (Figure 5). To our knowledge, this is the first evidence that two major antiaging factors are functionally and mechanistically connected during renal aging process. Previous studies have shown that oxidative stress suppresses KLOTHO (Song et al., 1999) and KLOTHO deficiency or reactivation inversely affects oxidative stress by acting on NRF2 signaling (Maltese et al., 2017). Together with our data, these observations suggest that KLOTHO and NRF2 suppressions are affected by aberrant DNMT elevations, which form a regulatory loop in a positive fashion to additionally control renal aging and the antiaging effects of DNA demethylation. The antiaging effects of NRF2 are mainly attributed to its beneficial regulation of oxidative stress and inflammatory responses (Swamy et al., 2016), whereas KLOTHO seemly affects renal aging through multiple signaling pathways and cellular processes, such as insulin/insulin‐like growth factor‐1 signaling, oxidative stress, inflammation, fibrosis, and apoptosis (Kurosu et al., 2005; Liu et al., 2011; Masuda et al., 2005; Seok‐Jo Kim et al., 2017), likely through both NRF2‐dependent (Maltese et al., 2017) and NRF2‐independent (Yamamoto et al., 2005) signaling pathways. Therefore, although deficiency of either KLOTHO or NRF2 individually reduces the anti‐renal aging effects by DNA demethylation, KLOTHO has a greater genotype effect and effect size of interactions between KLOTHO genotype and D‐gal treatment and OLP intervention (Figure 5b, P2/η^2^ and P3/η^2^), and is the key target of the epigenetic anti‐renal aging intervention.

The bioactive components from dietary food or medicinal plants are emerging as the rich sources of epigenetic drugs with tolerable side effects (Chistiakov et al., 2017). Several plant phenolic compounds, such as curcumin, epigallocatechin‐3‐gallate, and resveratrol, possess impressive antiaging and epigenetic modulating capacities (Casamenti & Stefani, 2017). An oleuropein analogue decarboxymethyl oleuropein aglycone is capable of inhibiting DNA methylation activity (Corominas‐Faja et al., 2018). The olive phenolic compounds have shown effective protective potencies against aging‐related diseases, including neurodegenerative disorders, atherosclerosis, and cancer (Casamenti & Stefani, 2017). OLP is the most prevalent phenolic component found in olive leave, seed, pulp, and peel with impressive anti‐inflammatory and anti‐oxidative stress activities (Ghanbari et al., 2012). We discovered that OLP inhibited the aberrant DNMT1/3a/3b elevations and DNMT sensitively recovered the KLOTHO and NRF2 losses in D‐gal‐treated renal cells. Experiments with kKLOTHO gene knockdown and NRF2 gene knockout mice further demonstrated that its regulations of KLOTHO and NRF2 suppression are critical for its antiaging functions. These data provide strong evidence that olive phenolic compounds contain potent antiaging activities and a significant part of which is attributed to its preservation of antiaging factors through a mode of DNA hypomethylation action.

In conclusion, we have demonstrated that aberrant DNMT1/3a/3b elevations and the resultant suppression of antioxidant aging suppressor KLOTHO and NRF2 promote renal aging, as DNA‐demethylating agent SGI‐1027 and OLP effectively reverse the epigenetic alterations and reduce the renal aging alterations in a KLOTHO and NRF2‐dependent manner in D‐gal mice, since synthetic demethylating compounds, such as decitabine (Ornstein et al., 2015), are not suitable for prophylactic use due to uncertain side effects and potential cytotoxicity. Future exploration of bioactive DNA‐demethylating components from dietary or medicinal plants might yield effective epigenetic strategies to delay or alleviate renal aging and the aging‐associated kidney disorders.

## 4 MATERIALS AND METHODS

### 4.1 Animal studies

The use of animals and the animal protocols complied with the ARRIVE guidelines, conformed to the European Directive 2010/63/EU, and were approved by the Institutional Animal Care Committee (IACUC) of Nanjing University. C57BL/6J and ICR mice were from Model Animal Research Center of Nanjing University. Mice were maintained under a standard environmental condition (25 ± 2°C; 12‐h light‐dark cycle) and allowed free access to water and regular sterile chow diets containing 20.6% protein, 12% fat, and 67.4% carbohydrate (SWS9102; Xietong Pharmaceutical Bio‐engineering Company).

Studies of natural renal aging were performed with young and old C57BL/6J mice of 2−25 months of age available in laboratory. The accelerated renal aging was induced by D‐galactose (D‐gal) treatment, which is a well‐established aging model in vitro and in vivo (Azman & Zakaria, 2019). The experiments were performed with three sets of mice, namely 8‐ to 10‐week‐old ICR male mice, ICR male mice treated with siRNAcontrol (siCtrl) or siRNAKLOTHO (si*Kl*), and NRF2 knockout male mice and the control littermates (Cai et al., 2015). The mice of each set were randomly divided into six groups (8 mice in each group): (1) vehicle control; (2) oleuropein (OLP, 50mg/kg daily by oral gavage) (Fiorella Casamenti & Stefani, ); (3) D‐gal aging mice (D‐gal subcutaneous injection, 500mg/kg daily) (Zhao et al., 2020); (4) OLP intervention (OLP‐treated D‐gal mice); (5) SGI‐1027 (SGI, intraperitoneal injection at 2.5 mg/kg daily) (Reyes‐Aguirre & Lamas, 2016); and (6) SGI intervention (SGI‐treated D‐gal mice). The effective dosages of OLP, D‐gal, and SGI were based on previously published studies and optimized in our preliminary investigations (data not shown). The experiment went on for 8 weeks.

For in vivo siRNA‐mediated mouse KLOTHO gene (*Kl*) knockdown, a small interfering RNA (siRNA) targeting *Kl* mRNA (5′ GCGACTACCCAGAGAGTA T‐3′, 10 nm in 200 μl of phosphate‐buffered saline; Genescript) was injected through tail vein one day before D‐gal treatment and then twice a week during the experimental period. The control siRNA contained a scrambled RNA sequence.

### 4.2 Renal histology and senescence‐associated β‐galactosidase (SA‐β‐Gal) staining

Paraffin‐embedded kidney sections were stained with Masson's trichrome to determine the fibrosis‐related collagen deposition as before (Zhang et al., 2017). For SA‐β‐Gal staining, the kidney sections (8 μm thickness) embedded in optimal cutting temperature compound were processed with a commercial kit (C0602; Beyotime Biotech) following the manufacturer's instructions. Images were captured with a light microscope (Olympus, Japan). The extents of fibrosis and positive SA‐β‐Gal signals were blindly assessed and calculated as the ratio of collagen deposition or β‐Gal‐positive areas over the whole field based on ten randomly selected non‐overlapping fields and averaged for each animal.

### 4.3 Immunohistochemical and immunofluorescent staining

Immunohistochemical (IHC) and Immunofluorescent (IF) staining of kidney sections was performed essentially as before (Zhang et al., 2017) with primary antibodies to KLOTHO (A12028; Abclonal), NRF2 (sc‐722; Santa Cruz), and CD68 (25747‐I‐AP; Proteintech) following routine procedures.

### 4.4 Cell culture

Human renal tubule epithelial HK2 cells and human embryonic kidney HEK293 cells (ATCC) were maintained in DMEM/F12 or DMEM medium (Hyclone), respectively, with 10% FBS at 37°C in a humidified 5% CO_2_ incubator. Cells were treated with D‐galactose (G0750; Sigma), OLP (JBZ‐0396; Jin Yibai BioTech), or SGI‐1027 (HY13962; MedChemExpress) as indicated.

### 4.5 Western blot analysis

Protein expression of renal tissues or cells was analyzed by Western blotting following a regular procedure. The primary and secondary antibodies used were as following: KLOTHO (A12028), γH2AX (phosphorylated histone H2AX, AP0099; Abclonal); NRF2 (sc‐722), α‐SMA (sc‐32251; Santa Cruz Biotech); DNMT1(ab188453), DNMT3b (ab79822; Abcam), DNMT3a (bs‐0497R; Bioss); GAPDH (60004–1‐Ig; Proteintech); goat anti‐rabbit IgG‐HRP and goat anti‐mouse IgG‐HRP (YFSA02 and YFSA01; Yifeixue Biotech).

### 4.6 Plasmid constructions and cell transfection

The plasmids overexpressing flag‐tagged DNMT1 (F‐Dt1) and DNMT3a (F‐Dt3a) were obtained from VectorBuilder. The plasmids were transfected into HEK293 cells with Lipofactamine 2000 (11668–019, Invitrogen, USA) according to manufacturer's instruction.

### 4.7 Quantitative real‐time PCR (qRT‐PCR)

Total RNAs from mouse kidneys or human renal tubular epithelial HK2 cells were isolated using a Total RNA Extraction kit (R401‐01; Vazyme) according to the manufacturer's instructions. Equal amounts of mRNA were reversely transcribed to cDNA using a HiScript RT SuperMix kit (R122‐01; Vazyme). Quantitative real‐time PCR was performed with ChamQ Universal SYBR qPCR Master Mix (Q711‐02; Vazyme) on a Viia 7 quantitative real‐time PCR instrument (Thermo‐Fisher Scientific). The primer sequences for TNF‐α (Mouse gene *Tnfa*, m*Tnfa*F and m*Tnfa*R; human gene *TNFA*, h*TNFA*F, and h*TNFA*R), IL‐6 (mouse gene *Il6*, m*Il6*F, and m*Il6*R; human *IL6*, h*IL6*F, and h*IL* *6*R), and the control GAPDH (mouse gene *Gapdh*, m*Gapdh*F, and m*Gapdh*R; human gene *GAPDH*, h*GAPDH*F, and h*GAPDH*R) were listed in the Table 1. For each detection, a 20 μl of reaction volume included 10 μl of master mixture, 2 μl of diluted cDNA, 0.6 μl each primer, and sterile distilled water. The mRNA levels were calculated using the $2^{‐\Delta \Delta C_{t}}$ method and expressed as relative fold changes.

**TABLE 1:** Primer sequences of qRT‐PCR, MSP, and BSP

PCR primer	Sequences
m*Tnfa*F	CATCTTCTCAAAATTCGAGTGACAA
m*Tnfa*R	TGGGAGTAGACAAGGTACAACCC
m*Il6*F	GAGGATACCACTCCCAACAGACC
m*Il6*R	AAGTGCATCATCGTTGTTCATACA
m*Gapdh*F	TATGTCGTGGAGTCTACTGGTGT
m*Gapdh*R	GTCATCATACTTGGCAGGTTTCT
h*TNFA*F	TGCACTTTGGAGTGATCGGC
h*TNFA*R	GGGCCAGAGGGCTGATTAGA
h*IL6*F	TGAGGAGACTTGCCTGGTGA
h*IL6*R	ATTTGTGGTTGGGTCAGGGG
h*GAPDH*F	AGGTGGTCTCCTCTGACTTC
h*GAPDH*R	CTGTTGCTGTAGCCAAATTCG

MSP primer	Sequences
m*Kl*‐MF	GGTATCGCGGGTATTTTTAATC
m*Kl*‐MR	CGACATAATCCCTAAAATAATCGAC
m*Kl*‐unMF	TTAATGGTATTGTGGGTATTTTTAATTG
m*Kl*‐unMR	CAACATAATCCCTAAAATAATCAAC
Inp‐m*Kl*F	TAGTTTTAGGAAGGTAAAGGGAGTG
Inp‐m*Kl*R	AAATCCCAAAAAAAACACAACAAA
h*KL*‐MF	AAAGAGAATGAATTTGAGCGTTTAC
h*KL*‐MR	ACTCCGCTAACAATAATTACCTACG
h*KL*‐unMF	AAGAGAATGAATTTGAGTGTTTATGA
h*KL*‐unMR	TCCACTAACAATAATTACCTACAAA
Inp‐h*KL*F	CCAACTCCAAATCCCCTCTCTAT
Inp‐h*KL*R	TGATTAATTTAGATTGGGTTTAGAGAAGGA
m*Nfe2l2*‐MF	GTTTTAAAGAGTTAGGGTTGGAGC
m*Nfe2l2*‐MR	AAATCAAATAAACTAAAATCGCACG
m*Nfe2l2*‐unMF	TGTTTTAAAGAGTTAGGGTTGGAGT
m*Nfe2l2*‐unMR	ATCAAATAAACTAAAATCACACAAA
Inp‐m*Nfe2l2*F	ATTCTGTTAGAGCTGTGCCCG
Inp‐m*Nfe2l2*R	GGATGAGTCCACGCTGCAAA

BSP primer	Sequences
Bis‐m*Kl*F	TTTTGTTTTTTATTGGAGATGTGG
Bis‐m*Kl*R	TCCCAATAATACAAAATAACCACC

### 4.8 Methylation‐specific PCR and bisulfite‐sequencing PCR

Prediction of CpG islands in *Kl* (gene name for mouse KLOTHO) and *Nfe2l2* (gene name for mouse NRF2) promoters and primer design for methylation‐specific PCR (MSP) and bisulfite‐sequencing PCR (BSP) were performed with the online software MethPrimer (http: //www.urogene.org/methprimer). Genomic DNA was isolated from kidneys or cells using the Animal Tissues/Cells Genomic DNA Extraction Kit (D1700; Solarbio), and then modified by bisulfate treatment and purified by the SanPrep Column PCR Product Purification Kit (B518141; Sangon Biotech). The mouse *Kl* promoter methylation on +594/+777 locus was assayed by MSP with methylated primer pair m*Kl*‐MF/m*Kl*‐MR (184 bp); unmethylated primers m*Kl*‐unMF/ m*Kl*‐unMR (188 bp); and input DNA control primers Inp‐m*Kl*F/Inp‐m*Kl*R (180 bp). The *KL* (gene name for human KLOTHO) promoter methylation (−315/−99 locus) of HK2 cells (human) was assayed with methylated primers h*KL*‐MF/h*KL*‐MR (216 bp), unmethylated primers h*KL*‐unMF/h*KL*‐unMR (213 bp), and Input DNA control primers Inp‐h*KL*F/Inp‐h*KL*R (153 bp). The promoter methylation of mouse NRF2 gene (*Nfe2l2*, −399/−257 locus) was assayed by MSP with methylated primers m*Nfe2l2*‐MF/m *Nfe2l2*‐MR (143 bp), unmethylated primers m *Nfe2l2*‐unMF/m *Nfe2l2*‐unMR (142 bp), and input DNA control primers Inp‐m *Nfe2l2*F/Inp‐m *Nfe2l2*R (144 bp). PCR products were analyzed on a 2% agarose gel and quantified by Image J software.

The *Kl* promoter methylation of mouse renal tissues was assayed by BSP with primers Bis‐m*Kl*F and Bis‐m*Kl*R (+466/+700, see Table 1), which amplified the same locus examined by MSP. Three randomly selected mice from control, D‐gal, SGI, SGI/D‐gal, OLP, or OLP/D‐gal group were subjected for BSP assay. The PCR products were separated by electrophoresis, and the target DNA fragments were purified and cloned into pGEM T Easy Vector (A1360; Promega). Five colonies from each mouse/PCR reaction were randomly chosen for sequencing, and the percentages of methylated cytosines over total cytosines within the cloned fragment were calculated.

### 4.9 Serum biochemistry

Measurements of blood urea nitrogen (BUN, D799850‐0100; Sangon Biotech) and serum creatinine (ab65340; Abcam) were performed with respective commercial assay kit, following manufacturer's assay protocols. In particular for creatinine assay, creatinine is converted by creatininase to creatine that is further converted to sarcosine, whose oxidized product reacts with a probe to generate red color (λ_max_ = 570 nm) and fluorescence (Ex/Em = 538/587 nm), which were recorded with a microplate reader.

### 4.10 Statistical analysis

All data are expressed as means ±SEM or box‐and‐whisker plots as follows: Midline represents median, box is the 25th‐75th percentiles, and whiskers are minimum and maximum. The data normal distributions and homogeneity test of variances were determined by Shapiro–Wilk test and Levene's test, respectively. The calculation of main effect (P) and effect size (large effect size, η^2^ ≥0.1379; medium effect size, 0.0588 ≤ η^2^ < 0.1379; small effect size, 0.0099 ≤ η^2^ < 0.0588; Cohen, 1988) and statistical analysis, including Student's *t* test, two‐way analysis of variance (ANOVA), or three‐way ANOVA followed by Tukey's post hoc test, were performed with SPSS V.22.0 software. Results were considered significant if the *p* values were <0.05.

## CONFLICT OF INTEREST

The authors declare no conflict of interest.

## AUTHOR CONTRIBUTIONS

Qi Gao performed the investigation, data analysis, and draft writing; Fang Chen, Lijun Zhang, Ai Wei, Yongxiang Wang, and Zhiwei Wu provided technique supports and research resources; Wangsen Cao designed the study, arranged the data, and wrote the manuscript. All authors have approved the final version of the manuscript.

## Supporting information

Fig S1

Fig S2

Fig S3

## Data Availability

The data that support the findings of this study are available from the corresponding author upon reasonable request.
