# Supplementary figures and images for: Inhibition of DNA methyltransferase aberrations reinstates antioxidant aging suppressors and ameliorates renal aging
Source: Aging Cell. 2021 Dec 7;21(1):e13526. doi: 10.1111/acel.13526 (PMC8761007; doi:10.1111/acel.13526)

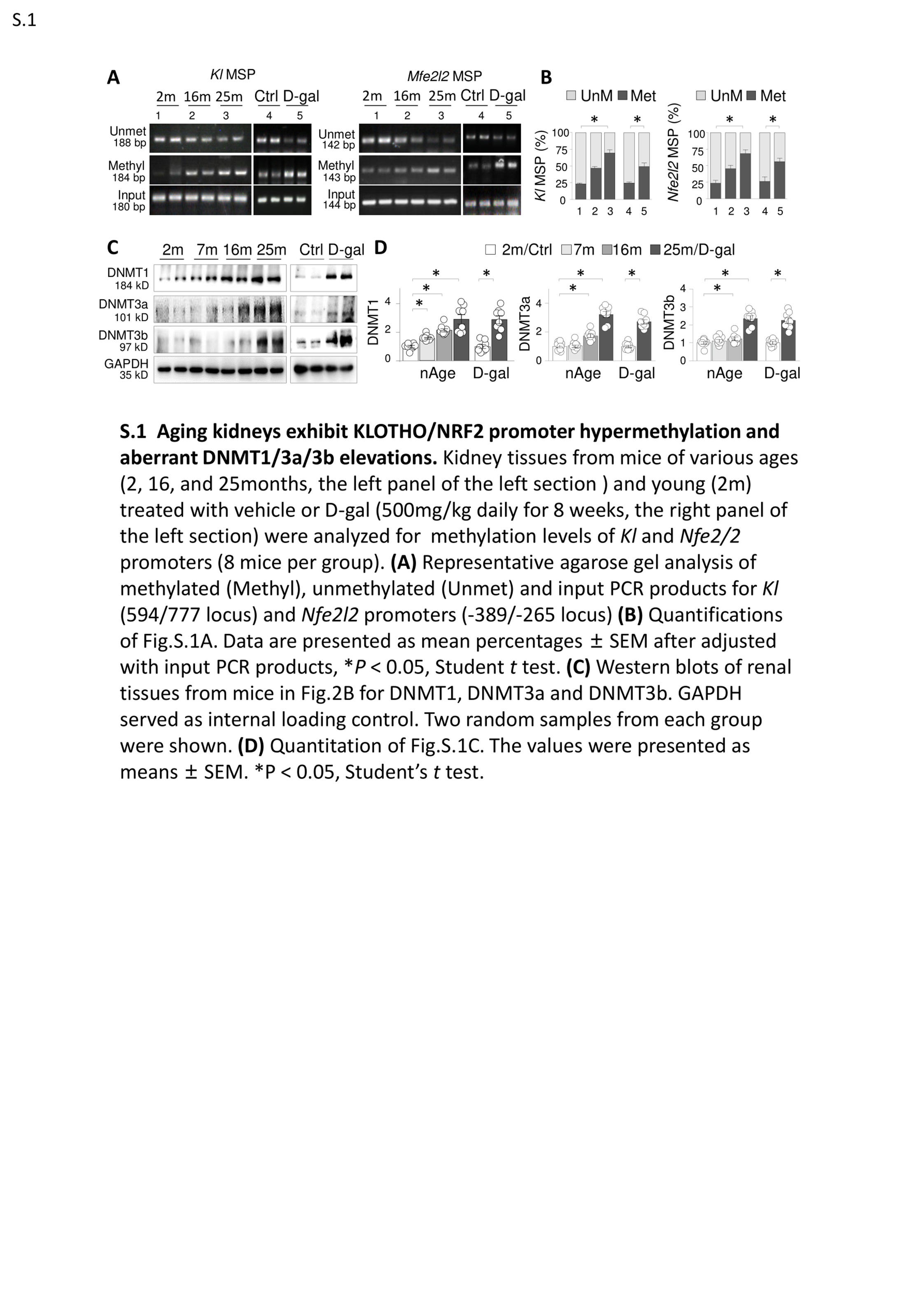

Supplement: Supplementary file 1 — Fig S1 [file ACEL-21-e13526-s003.tif]

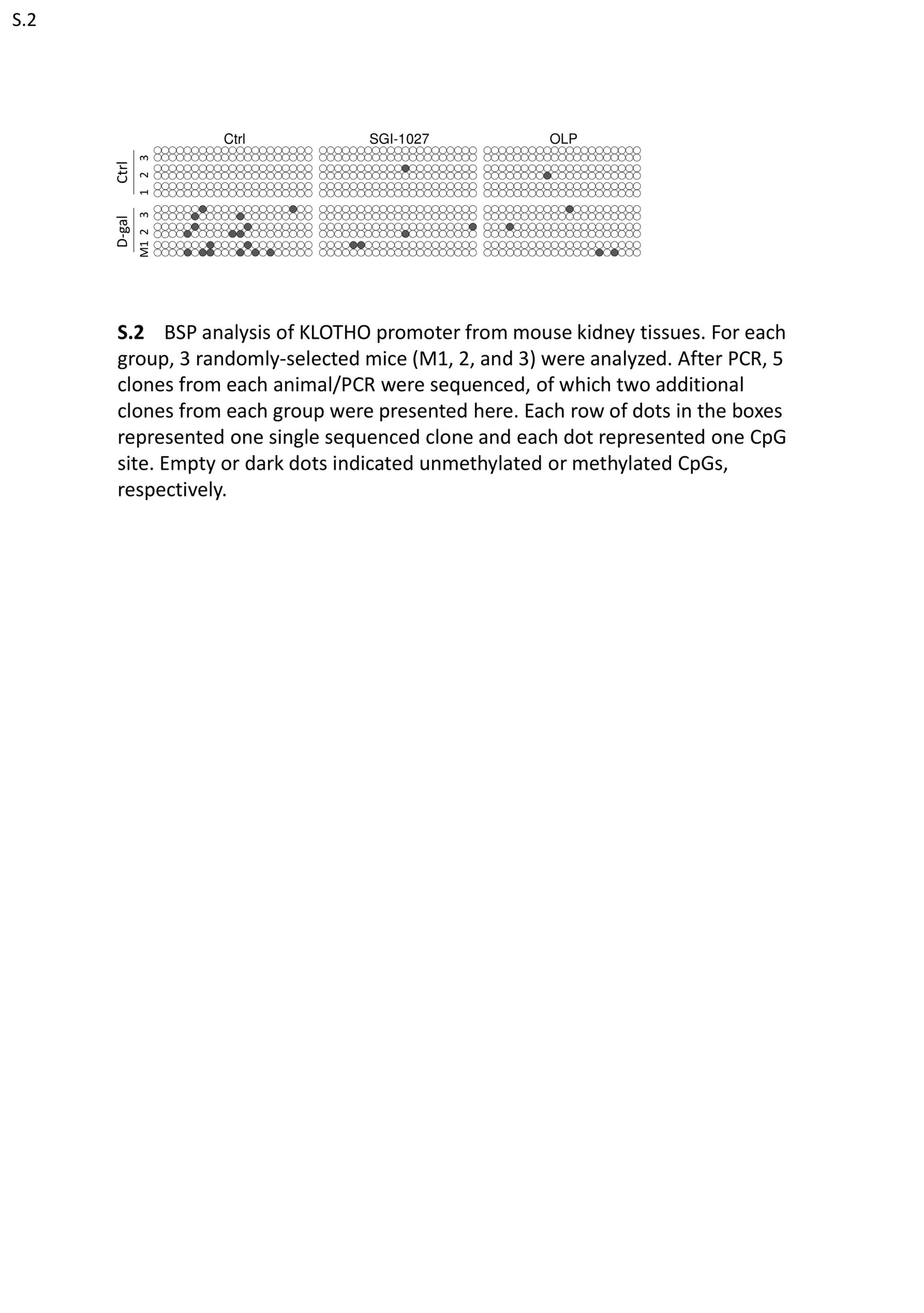

Supplement: Supplementary file 2 — Fig S2 [file ACEL-21-e13526-s002.tif]
